# Lactation Defect with Impaired Secretory Activation in AEBP1-Null Mice

**DOI:** 10.1371/journal.pone.0027795

**Published:** 2011-11-16

**Authors:** Lei Zhang, Shannon P. Reidy, Oleg Bogachev, Brian K. Hall, Amin Majdalawieh, Hyo-Sung Ro

**Affiliations:** 1 Department of Biochemistry & Molecular Biology, Dalhousie University, Halifax, Nova Scotia, Canada; 2 Department of Biology, Dalhousie University, Halifax, Nova Scotia, Canada; 3 Department of Biology, Chemistry and Environmental Sciences, Faculty of Arts and Sciences, American University of Sharjah, Sharjah, United Arab Emirates; University of Otago, New Zealand

## Abstract

Adipocyte enhancer binding protein 1 (AEBP1) is a multifunctional protein that negatively regulates the tumor suppressor PTEN and IκBα, the inhibitor of NF-κB, through protein-protein interaction, thereby promoting cell survival and inflammation. Mice homozygous for a disrupted AEBP1 gene developed to term but showed defects in growth after birth. *AEBP1* ^−*/*−^ females display lactation defect, which results in the death of 100% of the litters nursed by *AEBP1* ^−*/*−^ dams. Mammary gland development during pregnancy appears normal in *AEBP1* ^−*/*−^ dams; however these mice exhibit expansion of the luminal space and the appearance of large cytoplasmic lipid droplets (CLDs) in the mammary epithelial cells at late pregnancy and parturition, which is a clear sign of failed secretory activation, and accumulation of milk proteins in the mammary gland, presumably reflecting milk stasis following failed secretory activation. Eventually, *AEBP1* ^−*/*−^ mammary gland rapidly undergoes involution at postpartum. Stromal restoration of AEBP1 expression by transplanting wild-type bone marrow (BM) cells is sufficient to rescue the mammary gland defect. Our studies suggest that AEBP1 is critical in the maintenance of normal tissue architecture and function of the mammary gland tissue and controls stromal-epithelial crosstalk in mammary gland development.

## Introduction

The mammary gland is a self-renewing tissue in which morphological changes and differentiation occur cyclically during menstruation, pregnancy and lactation. In prepubescent mice, the gland consists of a small ductal tree that emanates from the nipple into the proximal part of the fatty stroma, the mammary fat pad. Upon initiation of ovarian hormone secretion, the mammary epithelium enters an accelerated growth phase that leads to extension and branching of the ducts until they reach the limits of the fat pad. At the onset of pregnancy, extensive epithelial cell proliferation occurs, leading to the formation of lobulo-alveolar structures and secretory epithelial differentiation for lactation. At parturition, a dramatic increase in the expression of milk proteins and lipid biosynthetic enzymes has been observed [1], [2]. The transition from late pregnancy to lactation is stimulated by a rise in prolactin and decrease in serum progesterone [3], and this transition is referred to as secretory activation [4], [5]. The event of involution that follows weaning results in the quenching of milk protein gene expression, collapse of the alveolar structures, removal of endothelial, myoepithelial and secretory luminal epithelial cells by apoptosis, phagocytosis by macrophages, proteolytic degradation of the basement membranes, and replacement of most epithelial cells by adipose tissue [6].

Adipocyte enhancer-binding protein 1 (AEBP1) is a transcriptional repressor that was initially shown to be involved in adipocyte differentiation [7]. Further study revealed that direct binding of the γ5 subunit of a heterotrimeric G protein with AEBP1 could regulate this transcriptional repression [8]. Aortic carboxypeptidase-like protein (ACLP) is an N-terminally extended, non-nuclear isoform of AEBP1 that contains a signal peptide and a lysine- and proline-rich 11-amino acids repeating motif [9], which are absent in AEBP1. Western blot analysis showed that ACLP is predominantly expressed in the smooth muscle cells of the adult mouse aorta but not in the adventitia, heart, liver, skeletal muscle, or kidney, while *in situ* hybridization experiments showed that ACLP is expressed in the smooth muscle cells of the aorta, but not in skeletal muscle cells [9]. In contrast, AEBP1 expression is detected in various tissues [10]. ACLP expression is up-regulated during vascular smooth muscle cell differentiation [9], whereas AEBP1 expression is down-regulated during adipocyte differentiation [7]. AEBP1 gene contains 21 exons spanning over 10 kb of genomic DNA. This gene gives rise to AEBP1 and ACLP mRNAs by alternative splicing [10].

AEBP1 has been found to interact with mitogen-activated protein kinase (MAPK) [11], suggesting an involvement in the signaling cascade directing extracellular signals to the nucleus. We have shown that MAPK regulates the transcriptional activity of AEBP1 by a novel dual mechanism, in which MAPK interaction enhances and subsequent phosphorylation decreases the DNA-binding ability of AEBP1 [12]. Using the yeast two-hybrid system, AEBP1 was also previously identified as an interacting partner of the tumor suppressor PTEN (phosphatase and tensin homolog deleted in chromosome ten) [13]. We have shown that AEBP1 physically interacts with PTEN in mammalian cells and this interaction promotes PTEN degradation [14]. Studies with AEBP1 transgenic (*AEBP1^TG^*) mice, which over-express AEBP1 transgene in the adipose tissues and macrophages [14], and *AEBP1* ^−*/*−^ mice [15] suggested that AEBP1 plays a key functional role in *in vivo* modulation of adiposity through its negative regulation of PTEN. PTEN has also been shown to play an important role in mammary gland development and tumorigenesis [16], [17].

AEBP1 is also a pivotal player in macrophage cholesterol homeostasis and macrophage inflammatory responsiveness; key events involved in atherogenesis [18], [19]. AEBP1 impedes macrophage cholesterol efflux and promotes foam cell formation via PPARγ1 and LXRα down-regulation, which is accompanied by concurrent repression of major cholesterol efflux mediators, leading to foam cell formation [20]. AEBP1 was shown to promote macrophage inflammatory responsiveness by inducing NF-κB activity through hampering IκBα inhibitory function via protein-protein interaction [21]. We further showed that LPS-induced down-regulation of pivotal macrophage cholesterol efflux mediators, leading to foam cell formation, is largely mediated by AEBP1, and this regulatory role of AEBP1 is physiologically relevant given that AEBP1 ablation attenuates LPS-induced foam cell formation [22]. Finally, our recent *in vivo* data clearly demonstrated that macrophage AEBP1 plays critical regulatory roles in atherogenesis [23].

In this study, we explore the physiological role of AEBP1 in the lactation defect observed in AEBP1-null mice. Our results highlight the role that AEBP1 plays in signaling physiological maintenance of normal tissue architecture and function of the mammary gland tissue. BM transplantation experiments reveal that stromal expression of AEBP1 is required for normal mammary gland development. Our results presented here show that AEBP1 is a critical stromal factor that mediates stromal-epithelial crosstalk in the regulation of mammary gland development.

## Materials and Methods

### Ethics Statement

All animal protocols used in these investigations have been reviewed and approved by the Dalhousie University Animal Care committee.

### Mouse strains

Generation of *AEBP1* ^−*/*−^ mice was previously described [15]. Age-matched mice were kept on a 12 hr light cycle in the Carleton Animal Care Facility at Dalhousie University where they were fed and watered *ad libitum*. Mice were sacrificed by euthanasia using an overdose of sodium pentobarbital (Somnitol), and mammary gland tissues were isolated for histological, whole mount and Western blot analyses.

### Histology and whole mount analysis

For the whole mount preparation of mammary glands, mice were sacrificed by cervical dislocation at different ages of virgin, at the appropriate time points during pregnancy, and at parturition and postpartum. The first inguinal and thoracic glands were removed and fixed in Carnoy's solution (ethanol:chloroform:glacial acetic acid, 6∶3∶1) for 4 hr. Subsequently, the glands were hydrated, stained with carmine-alum overnight, dehydrated, cleared in xylene, and mounted with permount. For histological analysis, mammary gland tissues were fixed in 10% acetate buffered formalin, paraffin embedded, sectioned at 5 µm and stained with hematoxylin and eosin. Histologic sections of mammary glands were examined in each instance.

### Immunohistochemistry

Mammary gland sections were deparaffined, rehydrated and treated in 10 mM sodium citrate (pH 6.0) by boiling for 10 minutes in microwave oven for antigen unmasking. The slides were treated with 3% hydrogen peroxide to inactivate endogenous peroxides and then treated with normal rabbit serum. The sections were treated with anti-p-STAT3 (Cell Signaling Technology, *Beverly, MA, USA*), anti-cleaved caspase 3 (Cell Signaling Technology), or anti-C/EBPδ (Santa Cruz Biotechnology, *Santa Cruz, CA, USA*). The immunoperoxidase staining was performed using Vectastain ABC kit (Vector Laboratories, *Burlingame, CA, USA)* according to the manufacturer's protocol. The signal was visualized using DAB peroxidase substrate (Sigma-Aldrich Co.).

### Mammary epithelial cell transplantation

Epithelial cell transplants were used to determine if AEBP1 protein is expressed in stromal or epithelial compartments of the mammary gland. Surgical removal of the parenchyma results in a gland-free mammary fat pad, referred to as cleared fat pad (CFP), which is suitable for receiving donor tissue. Briefly, portions of mammary gland (<2 mm^2^) were isolated from an adult donor mouse (≥ 6 weeks old) and transplanted into the inguinal (#4) CFP of a 3 to 4 weeks old female mouse. The transplanted mammary gland tissue grew to form chimeric secondary mammary glands consisting of donor-derived epithelial component and recipient-derived stroma. Three types of chimeras were generated: AEBP1^+/+^ donor tissue into AEBP1^+/+^ recipient mice, AEBP1^+/+^ into AEBP1^−/−^ recipient mice, and AEBP1^−/−^ donor tissue into AEBP1^+/+^ recipient mice. The thoracic #3 mammary glands of the recipients served as controls. The mice were allowed to recover for at least 3 weeks and were subsequently mated with wild-type males. Following delivery (parturition), the mice were euthanized and their #3 normal and #4 chimeric mammary glands were dissected out. Half of each gland was immediately frozen in liquid N_2_ for protein analysis while the other half was prepared for histological analysis. Rat mAb TROMA-1 (Developmental Studies Hybridoma Bank, University of Iowa) was used to detect keratin 8; an epithelial marker.

### Bone marrow transplantation

Recipient mice at the age of 8–10 weeks were provided with acidified water with antibiotic (neomycin, 2 mg/ml) for 2 weeks before BM transplantation procedure. BM cells were collected from the femurs and tibias of donor mice (6–8 weeks old). Total T cells were depleted using CD90.2 microbeads (EasySep, StemCell Technologies (Vancouver, BC, Canada). Recipient mice were lethally irradiated (900 Rad), and then injected with BM cells (5.0×10^6^) through tail vein. Two weeks after BM transplantation, mice were placed on high-fat diet (HFD) for 20 weeks or mated at 8 weeks post BM transplantation prior to whole mount analysis. Donor cell engraftment was evaluated by qRT-PCR analysis of SRY expression in the splenocytes and BM cells.

### Protein extraction and Western blot analysis

Mouse mammary gland whole cell protein extracts were prepared by homogenization in high-salt buffer (500 mM NaCl, 10 mM Tris [PH 7.4], 1% Triton X-100, 2 mM EDTA, 1 mM DTT, 0.1 mM PMSF, 1 mM Na_3_VO_4_, 1 mM sodium molybdate, and protease inhibitor cocktail). Mammary gland tissue was also digested in collagenase/dispase (Sigma) solution for 2 hr at 37°C. Separation of macrophages and epithelial cells were performed using magnetic EasySep Mouse Monocyte Enrichment Kit and EasySep Mouse Epithelial Cell Enrichment Kit (EasySep, StemCell Technologies, BC, Canada), and cell purity was confirmed by flow cytometry. For nuclear protein extraction, the Active Motif nuclear protein extraction kit (Active Motif) was used according to the manufacturer's recommendations. Cell lysis was performed on ice for 30 min. Clear protein extracts were obtained by centrifugation for 30 min at 4°C. Protein separation and immunoblotting, followed by ECL immunodetection system (Amersham), were performed as previously described [14].

### Statistical analysis

Data is expressed as the mean ± S.D. of the indicated number of samples. Statistical significance was determined using Student *t*-test for un-paired observations. *P<0.05 and **P<0.001 are considered statistically significant.

## Results

### AEBP1 is critical in mammary gland development

**Figure 1:**
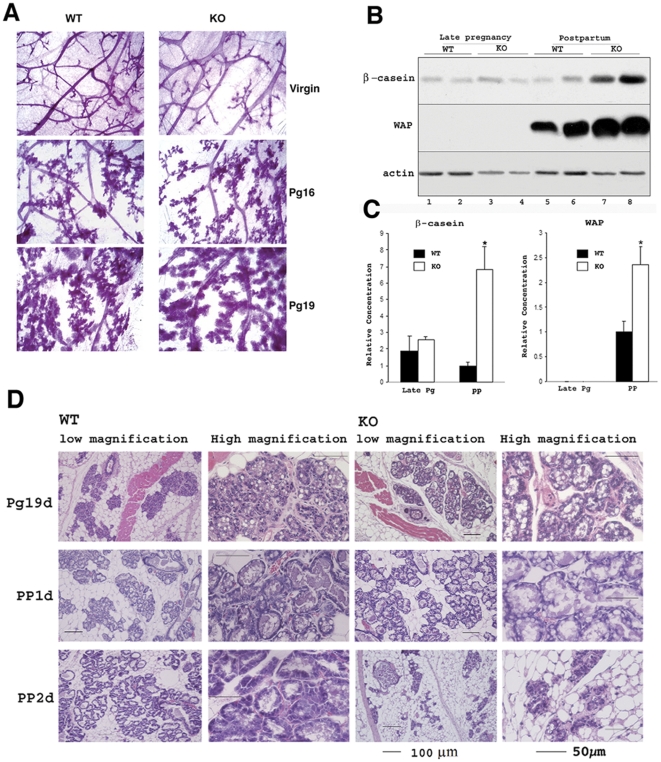
Mammary gland defect in *AEBP1* ^−*/*−^ mice. (**A**) Whole mounts of mammary glands isolated from *AEBP1^+/+^* and *AEBP1* ^−*/*−^ mice at different mammary gland developmental stages. (**B**) Expression of β-casein and WAP in *AEBP1^+/+^* and *AEBP1* ^−*/*−^ mammary glands at late pregnancy (lane 1, Pg18; lanes 2–4, Pg19) and postpartum (lanes 5, 7, 8, PP1d; lane 6, PP2d) was examined using anti-β-casein and anti-WAP antibodies, respectively. (**C**) Histograms illustrate densitometric analysis of protein levels shown in (B) where relative protein levels of β-casein and WAP were determined based on actin expression (*n* = 2–3). (**D**) Haematoxylin-eosin staining of *AEBP1^+/+^* and *AEBP1* ^−*/*−^ mammary glands at different mammary gland developmental stages.

Homozygous *AEBP1* ^−*/*−^ female mice display defective mammary gland development. When 6–8 weeks old *AEBP1* ^−*/*−^ females were mated with either *AEBP1^+/+^* or *AEBP1^+/^* ^−^ males, most of their pups died within 24 hr, with the entire litter dying by 48 hr. Of fifteen F_2_ *AEBP1* ^−*/*−^ mothers analyzed, all were unable to nurse their pups as determined by the lack of milk in the pup's stomachs, despite continued mothering and nurturing by the mothers and suckling by the pups. Upon fostering with hemizygous mothers, pups of *AEBP1* ^−*/*−^ mothers thrived. Whole mount analysis indicated that ductal branching and alveolar development that accompany pregnancy appear unimpaired in *AEBP1^−/−^* mice (**Fig. 1A**). Although mammary gland tissue of *AEBP1* ^−*/*−^ females appeared fully developed and filled the fat pad during late pregnancy, it was unclear whether the alveoli in these mice differentiated normally with parturition. Hallmarks of mammary epithelial cell differentiation are the transcriptional activation of milk protein genes by signal transducer and activator of transcription (STAT)5 via prolactin (Prl) signaling, followed by the synthesis and secretion of their corresponding proteins (β-casein and WAP) [24], [25]. As shown in **Fig. 1B**, β-casein and WAP proteins are detectable in *AEBP1* ^−*/*−^ mice during late pregnancy (Pg) and postpartum (PP), suggesting that AEBP1 is an obligate mediator of normal mammary gland function. Interestingly, both β-casein and WAP levels are significantly increased in *AEBP1* ^−*/*−^ mammary gland at PP (**Fig. 1C**). Accumulation of milk proteins in the mammary gland, which presumably reflects milk stasis, suggests failed secretory activation in the *AEBP1* ^−*/*−^ mammary gland. Histological change marking the transition from pregnancy to lactation is the alteration in the size and cellular distribution of lipid droplets. At mid-pregnancy, small cytoplasmic lipid droplets (CLDs) can be seen within luminal mammary epithelial cells. By the end of pregnancy, the CLDs increase dramatically in size. Following parturition, CLDs are smaller and localized to the apical surface of the alveolar epithelial cells [5]. It is interesting to note that in many genetically-engineered mice that exhibit lactation failure, large CLDs remain after parturition and can be observed on the first and sometimes even the second day of lactation [26]–[32]. Consistently, *AEBP1* ^−*/*−^ mammary glands also exhibit greatly enlarged alveoli with extended lumina containing many large CLDs within luminal mammary epithelial cells at 1-day PP (PP1d, **Fig. 1D**), suggesting that secretory activation has not occurred. Subsequently, AEBP1^−/−^ mammary glands exhibit groups of collapsed alveoli with reduced size and number at 2-day PP (PP2d, **Fig. 1D**), a precocious involution induced by milk stasis post-partum. Taken together, accumulation of milk proteins in the mammary gland, expansion of the luminal space, and the appearance of large CLDs strongly suggest a failure in secretory activation in the AEBP1^−/−^ mammary glands.

### AEBP1 deficiency induces premature involution

**Figure 2:**
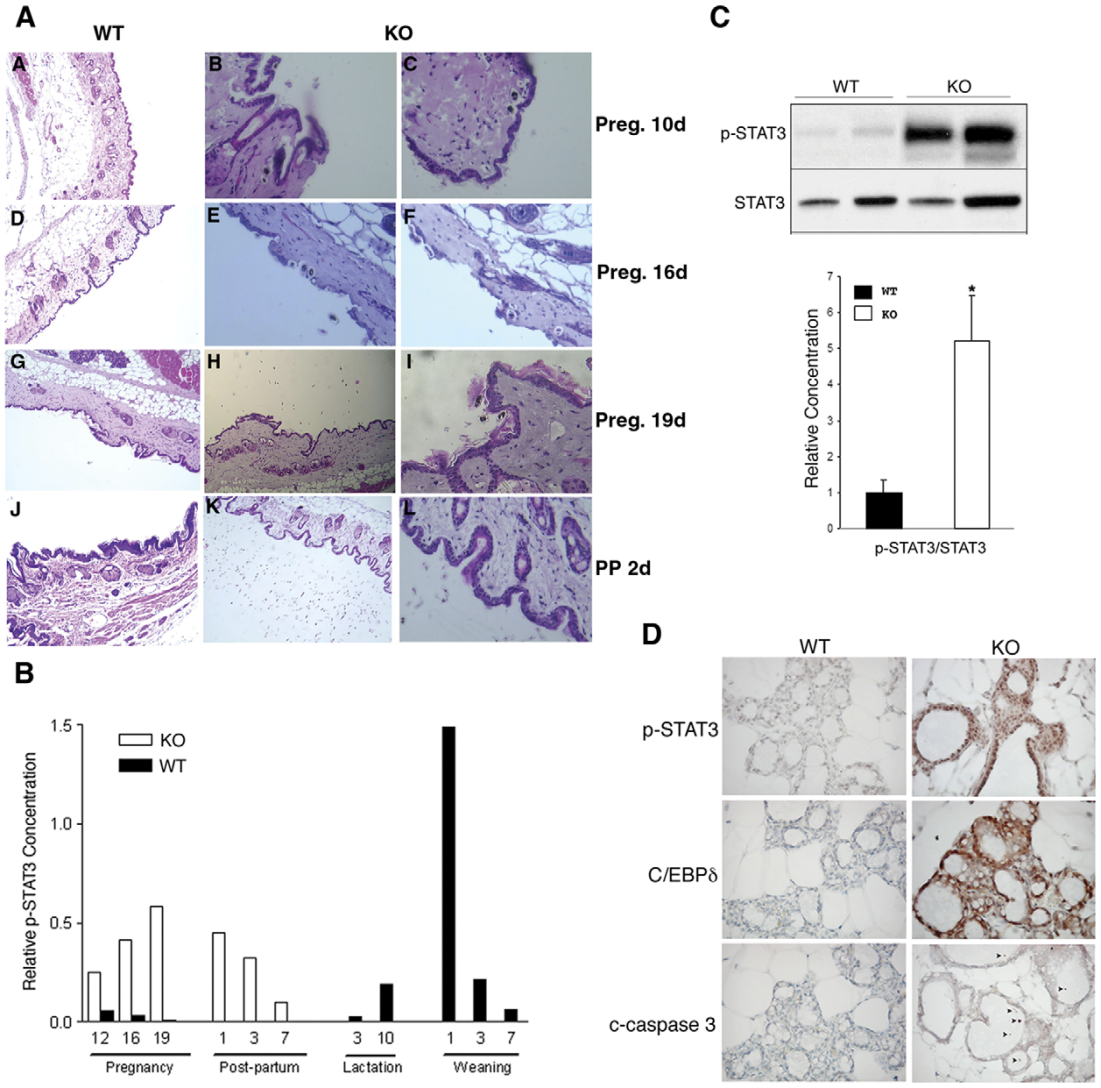
Ablation of AEBP1 causes progressive sloughing of cells and premature involution during pregnancy and a postpartum. (**A**) Haematoxylin-eosin staining of mammary glands during pregnancy (panels A to I) and at two days postpartum (panels J to L) from wild-type (WT) females (panels A, D, G, J) and *AEBP1* ^−*/*−^ (KO) females (panels B, C, E, F, H, I, K, L). Two different magnifications are shown for the KO samples. Panels H and K are in same magnification as the WT (panels A, D, G, J). (**B**) Expression of p-STAT3 protein (relative to total STAT3) in *AEBP1^+/+^* and *AEBP1* ^−*/*−^ mammary glands was examined using anti-p-STAT3 and anti-STAT3 antibodies. (**C**) Protein extracts isolated from *AEBP1^+/+^* (WT: lane 1, Pg16; lane 2, Pg19) and *AEBP1* ^−*/*−^ (KO: lane 3, Pg16; lane 4, Pg19) mammary glands were subjected to SDS-PAGE and immunoblotting using antibodies against the indicated proteins. A histogram illustrates densitometric analysis of protein levels where relative protein levels of p-STAT3 were determined based on total STAT3 (*n* = 3–5). (**D**) Sections from paraffin-embedded *AEBP1^+/+^* and *AEBP1* ^−*/*−^ mammary glands at Pg19 were stained with anti-p-STAT3, anti-C/EBPδ and anti-c-caspase 3 antibodies, and counterstained with hematoxylin. Arrows indicate apoptotic epithelial cells that have been shed into the alveolar lumen.

It is interesting that an excessive number of exfoliated cells were detected in *AEBP1* ^−*/*−^ mammary gland tissue (**Fig. 2A**). A small number of exfoliated cells were detected as early as day 10 of pregnancy (panels B, C). The number of exfoliated cells increased progressively during pregnancy (panels E, F, H, I), then a large number of the exfoliated cells were detected at 2-day PP (panels K, L). No such sloughing of cells was observed in wild-type tissues during this period (panels A, D, G, J). We further examined possible premature involution in *AEBP1* ^−*/*−^ mammary glands by comparing STAT3 phosphorylation (p-STAT3) status [33] and expression of CCAAT/enhancer binding protein (C/EBPδ) [34] and cleaved caspase (c-caspase) 3 [35] as markers of involution. Premature involution displayed by *AEBP1* ^−*/*−^ mammary glands at PP is evident based on p-STAT3 induction that usually peaks at day 1 of weaning (**Fig. 2B**). Surprisingly, p-STAT3 induction is evident as early as day 12 of pregnancy and its level is highest at day 19 of pregnancy in AEBP1^−/−^ mice. The amount of p-STAT3 in *AEBP1* ^−*/*−^ glands is about 5-fold higher than that in *AEBP1^+/+^* glands during late pregnancy (**Fig. 2C**). Immunohistochemical analysis also shows strong p-STAT3 signal in mammary epithelial cells of *AEBP1* ^−*/*−^ mice at day 19 of pregnancy (**Fig. 2D**). Similarly, intense C/EBPδ and c-caspase 3 signals are detected in *AEBP1* ^−*/*−^ mammary epithelial cells at day 19 of pregnancy (**Fig. 2D**). A number of possible apoptotic cells expressing c-caspase 3 are detected in the lumen of alveoli and within the epithelial cell layer of *AEBP1* ^−*/*−^ mammary glands (**Fig. 2D**). Circulating prolactin, a lactogenic hormone, levels are not affected by the disruption of AEBP1 gene in *AEBP1* ^−*/*−^ female/male mice (data not shown). These results suggest that AEBP1 is critical in signaling the initiation of physiological apoptosis during mammary gland involution.

### AEBP1 expression is modulated during the transition from pregnancy to lactation

**Figure 3:**
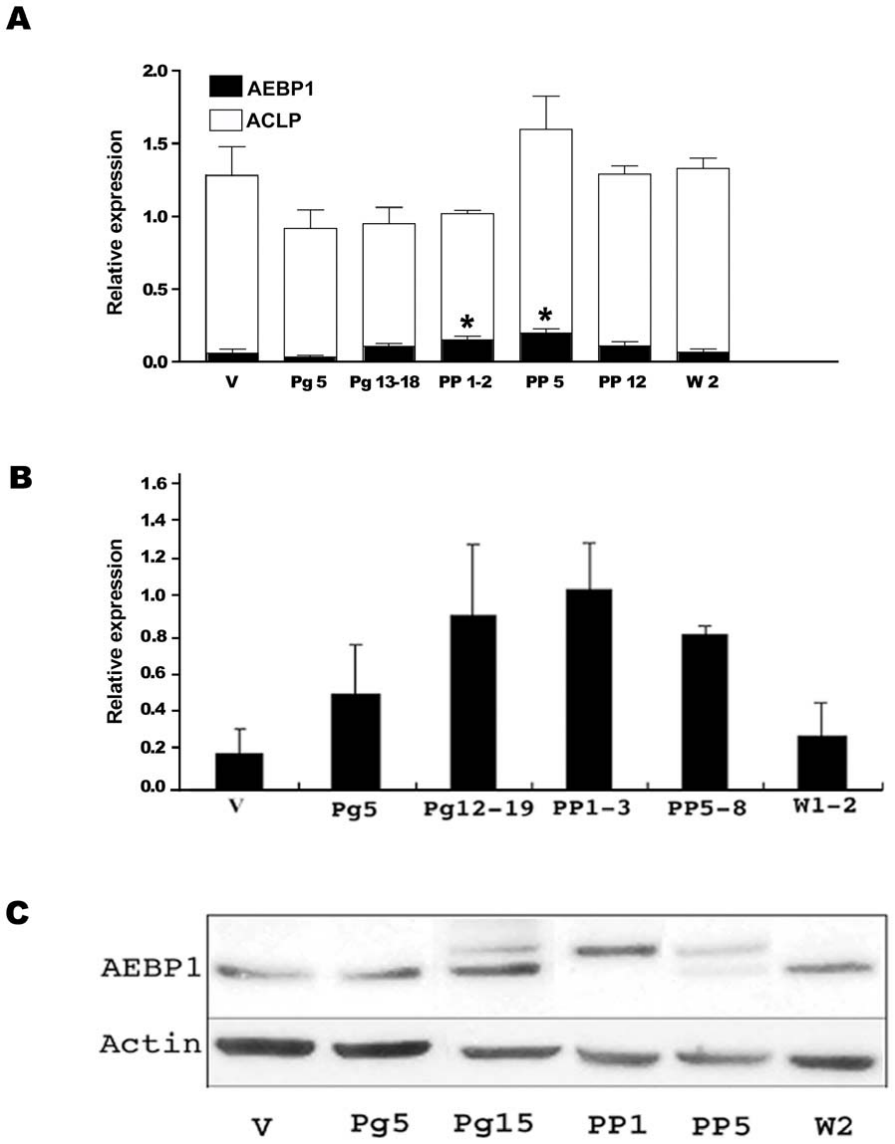
AEBP1 expression is regulated during mammary gland development. (**A**) Semi-quantitative RT-PCR analysis of AEBP1 and ACLP mRNA transcripts in mammary gland at various stages during postnatal development. AEBP1 and ACLP expression was normalized to the mRNA expression of the small subunit ribosomal protein S24. (**B**) AEBP1 protein levels at different stages were normalized based on actin expression (*n* = 5). (**C**) A representative Western blot analysis of AEBP1 expression at different stages of mammary gland development.

To further substantiate the role of AEBP1 in mammary gland development, we examined the expression profile of AEBP1 in mammary gland tissue at various stages during postnatal development. Based on the mammary gland defect found in *AEBP1* ^−*/*−^ dams, it is plausible to predict that AEBP1 expression is regulated during the transition from pregnancy to lactation when secretory activation occurs [4], [5]. Compared to those levels measured in virgin mice, AEBP1 mRNA expression increases slightly during late pregnancy (**Fig. 3A**). Initially during lactation, AEBP1 mRNA level further increases to about 4-fold above the virgin level. However, as the animal continues lactating, AEBP1 mRNA level drops. AEBP1 mRNA level also changes when the animal is weaned as it eventually decreases to the virgin level at day 2 of weaning. In contrast, ACLP mRNA (**Fig. 3A**) level was not significantly changed during mammary gland development. Compared to its level in virgin mice, AEBP1 protein level also increases during pregnancy (**Fig. 3B**). During early lactation (PP1-3), AEBP1 protein level further increases to about 5-fold higher than its level in virgin mice. However, as lactation continues, AEBP1 protein level significantly drops, and it continues to decrease at weaning (**Fig. 3B**). Gene expression analysis and immunoblot analysis demonstrate that expression of AEBP1 is indeed regulated at the stage of secretory activation during mammary gland development. Interestingly, there are two forms of AEBP1 protein during late pregnancy, in which the slow migrating form of AEBP1 is predominant at the beginning of the lactation period (**Fig. 3C**). During weaning, however, the fast migrating form of AEBP1 becomes more abundant than the slow migrating form (**Fig. 3C**). These results suggest that AEBP1 is post-translationally regulated during mammary gland development. Currently, the mechanism and functional significance of this modification are unknown. Together, our results suggest that AEBP1 is critically involved in mammary gland development during steady-state, pregnancy and postpartum stages. Determination of the localization of AEBP1 in mammary gland tissue (stromal vs. epithelial) could provide some hints regarding the molecular mechanism by which AEBP1 exerts its effects on mammary gland development.

### AEBP1 is a stromal factor and macrophage AEBP1 restoration rescues the mammary gland defect in *AEBP1^−/−^* mice

**Figure 4:**
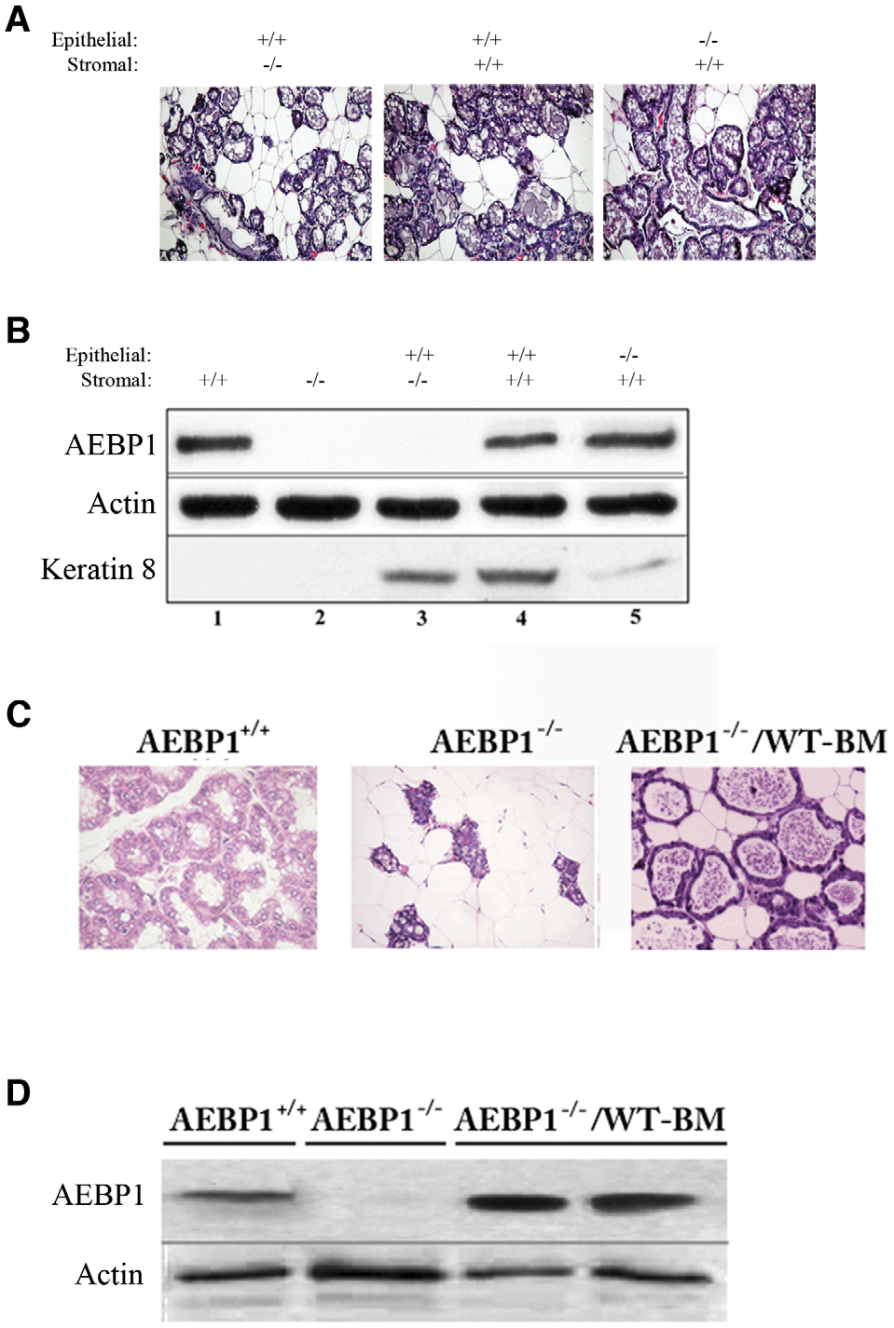
Stromal expression of AEBP1 regulates mammary gland development. (**A**) Haematoxylin-eosin staining of chimeric mammary glands at parturition. (**B**) Western blot analysis of AEBP1 expression in chimeric mammary glands (*n* = 3–5). Lane 1, CFP of *AEBP1^+/+^*; lane 2, CFP of *AEBP1* ^−*/*−^; lane 3, *AEBP1^+/+^* donor epithelial cells grown in CFP of *AEBP1* ^−*/*−^ recipient mouse; lane 4, *AEBP1^+/+^* donor epithelial cells grown in CFP of *AEBP1^+/+^* recipient mouse; lane 5, AEBP1^−/−^ donor epithelial cells grown in CFP of *AEBP1^+/+^* recipient mouse. Actin and epithelial marker keratin 8 were used as controls. (**C**) Restoration of stromal AEBP1 expression by BM transplantation (*AEBP1* ^−*/*−^/BM-WT chimeric mice) prevents premature involution displayed by *AEBP1* ^−*/*−^ mice at PP2d as determined by histology following hematoxylin and eosin staining. (**D**) Western blot analysis of AEBP1 expression in *AEBP1* ^−*/*−^/BM-WT mammary gland tissues.

Mammary epithelium grows in association with stroma, in which they communicate with each other through the extracellular matrix. Impaired mammary gland development in *AEBP1^−/−^* mice could be due to defects in stromal cells and/or a cell-autonomous defect localized to the epithelial cells. We have previously shown that AEBP1 is expressed in preadipocytes, but not in mature adipocytes [10]. AEBP1 is also abundantly expressed in macrophages [20]–[22]. Yet, it is difficult to detect AEBP1 in mammary stroma by immunohistochemistry possibly due to interference of adipocytes and/or unsuitability of the antibody to detect the endogenous level. Therefore, we carried out epithelial cell transplantation experiments [36]–[38] to determine the localization of AEBP1 in mammary gland tissue. To separate stromal from epithelial signals, *AEBP1^+/+^* or *AEBP1^−/−^* nulliparous mammary epithelial tissues were transplanted into cleared mammary fat pads of nulliparous *AEBP1^+/+^* or *AEBP1^−/−^* females. After formation of chimeric secondary mammary glands, which consist of donor-derived epithelium and recipient-derived stroma, mice were mated with wild-type males. Since *AEBP1^−/−^* mammary glands display alveolar collapse and epithelial cell hypoplasia at PP2d, the chimeric mammary glands were examined at parturition, exhibiting normal lobuloalveolar structure (**Fig. 4A**). Western blot analysis of the chimeric mammary gland tissues demonstrates that mammary epithelial cells do not express AEBP1 (**Fig. 4B**). AEBP1 expression is detected in the chimeric mammary gland tissue consisting of *AEBP1^+/+^* stroma (lanes 4 and 5), but not *AEBP1^−/−^* stroma (lane 3). Since AEBP1 expression was analyzed at PP, it is likely that the slow migrating form of AEBP1 found exclusively in the PP stage (**Fig. 3E**) corresponds to AEBP1 protein. Keratin 8, an epithelial marker, is expressed in all chimeric mammary gland tissues (lanes 3–5), but not in the cleared mammary fat pads (lanes 1 and 2). These results indicate that AEBP1 is expressed in the stromal compartment of mammary gland tissue, suggesting that stromal AEBP1 signals the crosstalk between mammary stroma and epithelium during mammary gland development.

Macrophages are an important component of mammary stroma and they are critical for normal mammary gland development [39]–[41]. However, the exact mechanisms underlying the role of macrophages in this process are poorly understood. Mammary macrophages are derived from circulating monocytes, and it is possible to repopulate mammary gland macrophages by BM transplantation following γ-irradiation [40]. Since AEBP1 is abundantly expressed in macrophages [20]–[22], we assessed whether stromal restoration of AEBP1 expression by transplanting wild-type BM cells could rescue the mammary gland development defect in *AEBP1^−/−^* mice. Remarkably, when the irradiated chimeric female mice were mated, all of their pups thrived. Histological analysis at PP2d reveals that mammary glands of the chimeric mice exhibit enlarged alveoli with extended lumina as in wild-type mammary glands (**Fig. 4C**). Western blot analysis demonstrates that AEBP1 levels are restored in the chimeric mammary gland tissues (**Fig. 4D**). These results strongly suggest that macrophage AEBP1 is a critical stromal factor mediating the stromal-epithelial crosstalk for functional development of the mammary gland.

## Discussion

To gain insight into the physiological activity of AEBP1, AEBP1-deficient mice were generated by homologous recombination. Mice homozygous for a disrupted AEBP1 gene developed to term but showed defect in growth after birth with no other macroscopic physical aberrations. *AEBP1* gene gives rise to *AEBP1* and *ACLP* mRNAs by alternative splicing [10]. Therefore, AEBP1-knockout mice, in theory, should be identical to the ACLP-knockout mice [42], as both disrupt the *AEBP1/ACLP* gene and the expression of both proteins. Mice lacking *ACLP* die perinatally due to gastroschisis, and only ∼6% of ACLP-knockout mice survive to adulthood [42]. Similarly, very small number of AEBP1-knockout mice survives to adulthood (<10%). The ratio of the three classes of mice from heterozygote mating indicated that the litter size of *AEBP1* ^−*/*−^ mice is about half of the wild-type [15], which suggests that *AEBP1* gene disruption also affects embryonic development. We have not characterized the embryonic lethality in the AEBP1-knockout mice. The predominant phenotypes of surviving adult *AEBP1* ^−*/*−^ mice are the inability of females to lactate due to defective secretory activation, the infertility of males possibly due to a fluid reabsorption defect in the efferent ductules of the reproductive system (unpublished observation), and an overall smaller size. Layne and coworkers never described the phenotypes of the surviving adult ACLP-knockout mice, except the deficiency in wound healing [42]. They concluded that ACLP is an extracellular matrix protein essential for normal embryonic development and dermal wound healing. The hypoproliferation phenotype of *ACLP* ^−*/*−^ fibroblasts observed in their studies is most likely due to AEBP1 disruption. We have previously shown that AEBP1 stimulates cell growth and survival and inhibits apoptosis through positive regulation of MAPK [11] and negative regulation of PTEN [14], [15]. AEBP1 was also shown to modulate MAPK activation by protecting it from the effects of a MAPK-specific phosphatase, suggesting that the modulation of MAPK activation by the protective interaction may constitute a critical role in the determination between cell growth and differentiation in the adipogenic lineage [11]. Cell fractionation and immunofluorescent staining experiments revealed that ACLP is excluded from the nucleus and cytosol and is localized in the perinuclear space, indicative of its entry into the secretory pathway [9]. Unlike ACLP, AEBP1 is detectable in the nucleus and cytosol [8]. Because ACLP is not localized to the cytosol, it is inconceivable that ACLP can have any effect on MAPK and PTEN functions by direct protein-protein interaction [14], [15], despite the fact that both AEBP1 and ACLP share the exact regions that mediated these interactions. AEBP1 also physically interacts with endogenous IκBα in macrophages, which do not express ACLP, and the interaction is physiologically relevant [20]–[22].

AEBP1 may be very critical in the functional development of the mammary gland. In *AEBP1* ^−/−^ females, the mammary epithelial compartment showed aberrant development at late pregnancy and female mice were unable to nurse their newborn pups, possibly due to an impaired secretory activation at parturition. The glands may have failed to switch to a lactation state resulting in a rapid involution postpartum. It appears that disruption of *AEBP1* gene caused premature mammary gland involution, which is characterized by elimination of epithelial cells by apoptosis. AEBP1 expression may be critical in regulating the apoptotic signal in mammary gland involution. The mammary gland has emerged as a rich developmental model that depends on epithelial-stromal interaction. The stroma-derived mediator that signals epithelial cell proliferation and apoptosis is still elusive. Crosstalk between the mammary stroma and epithelium is crucial for proper patterning and functioning of the mammary gland. Disruption of stromal-epithelial communication can promote uncontrolled proliferation of mammary epithelial cells and promote mammary tumorigenesis [43], [44]. Moreover, the exact composition of the extracellular matrix and the nature of the microenvironment surrounding mammary glands play critical roles in mammary gland morphogenesis, homeostasis and malignant transformation [45]–[48]. In this study, we present experimental findings suggesting a regulatory role for *AEBP1* in the control of mammary gland development, presenting AEBP1 as a novel developmental factor whose expression is vital for the maintenance of mammary gland homeostasis. Hence, AEBP1 is a critical stromal factor implicated in mammary epithelial cell growth. In addition, AEBP1 may function as an indispensable mediator of the communication signal between the mammary gland stromal and epithelial compartments, orchestrating the functional differentiation and involution of the mammary gland epithelium.

These recent discoveries and developments have changed our understanding of AEBP1 functions and have offered new insights into the mechanisms by which endogenous AEBP1 exerts its physiological effects. The specific lesions resulting from the absence of ubiquitously expressed *AEBP1* gene in only a few cell types may indicate combinatorial regulatory mechanisms that utilize shared components leading to unique responses in mammary gland tissue. Our results open up a new avenue of research directed towards further understanding of the regulation of mammary gland development. The availability of AEBP1-knockout and AEBP1-overexpressing transgenic mice will allow a more precise clarification of the role of AEBP1 in maintaining homeostasis in the mammary gland tissue.
