# Correction: Lactation Defect with Impaired Secretory Activation in AEBP1-Null Mice

**DOI:** 10.1371/annotation/4056d03c-20ed-4eca-9568-3e9400e2312e

**Published:** 2011-12-27

**Authors:** Lei Zhang, Shannon P. Reidy, Oleg Bogachev, Brian K. Hall, Amin Majdalawieh, Hyo-Sung Ro

The name of the fifth author in the citation line contained an error. The correct citation is: Zhang L, Reidy SP, Bogachev O, Hall BK, Majdalawieh A, et al. (2011) Lactation Defect with Impaired Secretory Activation in AEBP1-Null Mice. PLoS ONE 6(11): e27795. 

